# A new score for tomographic opacificacation of paranasal sinuses in children

**DOI:** 10.1590/S1808-86942010000400014

**Published:** 2015-10-19

**Authors:** Severino Aires de Araújo Neto, Emílio Carlos Elias Baracat, Leonardo Franco Felipe

**Affiliations:** a Master's degree, coordinator of the Image Diagnosis Module of the Paraiba Medical School (Faculdade de Ciências Médicas da Paraíba); b Doctoral degree, assistant professor, Pediatrics Department, UNICAMP Medical School; c Specialist, medical radiologist

**Keywords:** pediatrics, radiology, sinusitis, tomography

## Abstract

Many score methods have been created to measure paranasal sinus abnormalities seen under CT scan. Currently, the Lund-Mackay staging system is widely accepted. However, its results may be affected by the development in children.

**Aim:**

To assess the precision and accuracy of a new tomography score, called “opacification-development ratio”. It translates the percentage of sinus area that is opaque.

**Materials and Methods:**

A cross-sectional study was prospectively conducted in patients ranging from 0-18 years of age who underwent CT scan assessment of rhinosinusitis. Two independent radiologists examined each scan twice, using both the Lund system and the ratio herein proposed.

**Results:**

The opacification-development ratio reached substantial intra and inter-examiner agreement, similar to the Lund system (Kappa > 0.60). Considering the Lund system as the gold standard, the most accurate cut-off point was approximately 15 (sensitivity and specificity approach 90%). There was a strong linear correlation between the two methods (r > 90).

**Conclusions:**

opacification-development ratio is precise and correlates with the Lund system. A cut-off point set at 15 could be used to call a test positive.

## INTRODUCTION

Since the 1980s, computed tomography (CT) has been recommended as the gold standard for assessing rhinosinusitis (RS);^1^, ^2^, ^3^ it is an important component in the routine approach to the chronic form of this disease.^4^ The advantages of CT are its high sensitivity for inflammation (opacification)^5^ of the paranasal sinuses (PNS) and ability to demonstrate in detail the bony labyrinth of this area, including the narrow drainage pathways of the ostiomeatal complex (OMC).^6^ Thus, at the same time it discards other conditions that simulate chronic rhinosinusitis (CRS), CT may reveal eventual structural obstructive factors4 that help maintain the clinical picture.^7^

With the advent of functional endoscopic surgery (FES), CT has become as a “map” for surgeons by demonstrating the anatomy and its variants, and the distribution of opacification.^4^^,^^7^^,^^8^ In effect, there has been a search for measurement tools to translate the amount of rhinosinusal opacification into numbers or degrees at CT - the so-called scores.^9^, ^10^, ^11^, ^12^, ^13^, ^14^, ^15^ In objectively quantifying sinus opacification, scores have made it easier to correlate CT with clinical and endoscopic parameters, which may potentially help select patients that would benefit most from FES.^8^^,^^16^^,^^17^

In 1997, following a comparative analysis of eight better known scores, the American Academy of Otorhinolaryngology and Head & Neck Surgery, recommended the Lund-Mackay system (LMS).^18^ The LMS consists of checking 0 to 2 points for each cavity (maxillary, frontal, sphenoid, anterior ethmoid and posterior ethmoid) and for the OMC, that is, six sites in each side. Zero means a normal sinus, 1 (one) partial opacification, and 2 (two) complete opacification. The results is the sum of scores for each side, which ranges from zero to 24.^12^

The LMS has been proved to be practical and accurate in subsequent studies.^16^^,^^18^, ^19^, ^20^ However, it has not correlated regularly with the intensity of preoperative symptoms or the degree of clinical improvement after FES.^8^^,^^17^^,^^21^, ^22^, ^23^ One of its caveats is that the LMS groups together any partial opacification under a single score (one), thereby attributing equal values to both the presence of fluid and mucous thickening, which have different clinical outcomes.^24^^,^^25^ Furthermore, studies of scores have been made mostly in adults,^8^^,^^16^^,^^17^^,^^19^, ^20^, ^21^ and appear not to have taken into account the effect of absent sphenoid and frontal sinuses - a common feature of patients under 12 years of age - on their results. Given that the LMS is based on the sum of scores for each rhinosinusal compartment, absent (undeveloped) sphenoid and frontal sinuses reduce the score amplitude to 16 from 24 points, which results in artificially underestimating disease and therefore a bias when applied to children.

It is hoped that a tomographic score expressing an estimate (or percentage) of the proportion of opacification within cavities may be less prone to developmental interferences, and therefore applicable indistinctly to any age group. Such as score, named the opacification/development ratio (ODR), was used by the authors in a previous study of asymptomatic children and adolescents.^26^ This study aimed to verify the precision and accuracy of ODR and to compare it with the LMS, for validation.

## SUBJECTS AND METHODS

### Study Design

A contemporary cross-sectional cohort study to assess the precision and accuracy of a diagnostic tool.

### Subjects

An evaluation was made of the exams of patients aged from 2 to 18 years with a clinical diagnosis of RS that had been referred to the radiology unit for CT of the PNS from April 2002 to July 2004. Clinical diagnostic criteria, classification and intensity of disease were not arbitrated, as these exams served only as samples for repeated measures of sinus opacification on CT. Subjects with technically imperfect exams that did not permit an adequate appraisal of all PNS were excluded. Only the first exam was included in patients with two exams.

## METHODS

Exams were carried out using a Toshiba X-vision (Toshiba, Tokyo, Japan) device; sequential 1 to 2 mm coronal sections, eventually associated with axial sections, were done; no endovenous contrast media were used. Children under the age of 4 years were generally anesthetized for the exam; these patients were placed in dorsal decubitus, with the neck hyper-extended, and the gantry angulated to keep the coronal plane as the reference. No exam had its technical or operating conditions altered because of this study. The images were recorded in 1500-2500 opening and 100-400 level windows.

The ODR (Frame 1) separately assesses development and opacification. All sinuses and OMCs are considered as pairs, one for each side. The Frame below shows development scores for each sinus as 3 (three) if present and 0 (zero) if absent. The sum of five sites (4 cavities and the OMC) may reach 15 points for each side (30 in total) if all sinuses are developed. For opacification (see column in the Frame below), each cavity scores from 0 to 3 according to the opacified area: normal = 0 (zero); < 2/3 = 1 (one); ≥ 2/3 = 2 (two); total = 3 (three). Here, the OMC scores zero if normal, or 3 if opacified. Complete opacification of all sites in both sides adds up to 30 points. ODR calculation consists of the ratio: “sum of right and left opacification” (numerator) / “sum of right and left development” (denominator). The results ranges from 0 (normal) to 1 (complete opacification of the developed area). When multiplied by 100, it yields an estimated percentage of the global opacified area. The LMS was applied as previously described.^12^^,^^18^

**Frame 1:** Formula for the opacification/development ratio (ODR).

	RIGHT		LEFT	
	Development	Opacification	Development	Opacification
OMC	(0 or 3)	(0 or 3)	(0 or 3)	(0 or 3)
Maxillary	(0 or 3)	(0 to 3)	(0 or 3)	(0 to 3)
Ethmoid	(0 or 3)	(0 to 3)	(0 or 3)	(0 to 3)
Sphenoid	(0 or 3)	(0 to 3)	(0 or 3)	(0 to 3)
Frontal	(0 or 3)	(0 to 3)	(0 or 3)	(0 to 3)
Sums	(R.D.^a^)	(R.O.^b^)	(L.D.^c^)	(L.O.^d^)
ODR	(OD + OE) / (DD + DE) × 100*			

OMC: ostiomeatal complex; a: right development; b: right opacification; c: left development; d: left opacification. *ODR: the end value is given by the ratio “sum of right and left opacification” (numerator) / “sum of right and left development” (denominator). Ranges from 0 to 1 and indicates the percentage of opacified area when multiplied by 100.

Based on the two methods (ODR and LMS), two radiologists analyzed the images twice, totaling four sessions, separated by at least two weeks. Examiners had no access to each other's results or to their own first assessments.

After verifying the mean values of four LMS readings (two from each examiner), the sample was classified into opacification categories (normal, mild, moderate or severe), where 0 (zero) was normal, 0 to 3 was mild, 4 was 10 was moderate, and over 10 was severe. This division was based on studies by Bhattacharyya and Fried16 who, based on an accuracy analysis, defined an LMS ≥ 4 as an appropriate cut-off point for defining a positive CT for RS. In this same paper, the group of patients with clinically diagnosed CRS had a mean LMS score ≈ 10 (Frame 2). A preliminary analysis of the sample distribution revealed that each of these four categories (normal, mild, moderate or severe) contained about 25% of the sample (quartiles). Thus, quartile points on the scale were set to extract equivalent ODR borderline values; values > 15 were moderate, and values > 50 were severe (Frame 2).

**Frame 2:** Classification parameters of the sample by categories for each score

Category	LMS	ODR
Normal	0	0
Mild	1 a 3	1 a 14
Moderate	4 a 10	15 a 50
Severe	> 10	> 50

LMS: Lund-Mackay system; ODR: opacification/development ratio

This study design was approved by a CONEP-registered institutional review board, and registered under the protocol number 0814.0.146.000-08. A free informed consent form was made available beforehand to patients or their caretakers to authorize their participation in the study.

The Statistical Package for the Social Sciences (SPSS version 13.0; SPSS Inc., Chicago, IL, USA) was used for the statistical analysis. Method accuracy (reproducibility) was assessed using the intra and interobserver agreement Kappa coefficient (k) for categorical variables. Intra and interclass coefficients (intra-CC and inter-CC) were applied to assess agreement among quantitative variables (numerical scales of scores). Pearson's coefficient was applied to verify linear correlation between scales of both methods. ODR accuracy was extracted taking the LMS as the gold standard, with receiver operating characteristic (ROC) curve analysis.

## RESULTS

There were 81 exams from different patients; 17 were excluded because of incomplete images or artifacts. Of the 64 remaining exams, 38 were from males (60.3%). Ages ranged from 2 to 18 years (mean – 10 years, SD = 4). Two patients had undergone prior rhinosinusal surgery.

**Chart 1:**
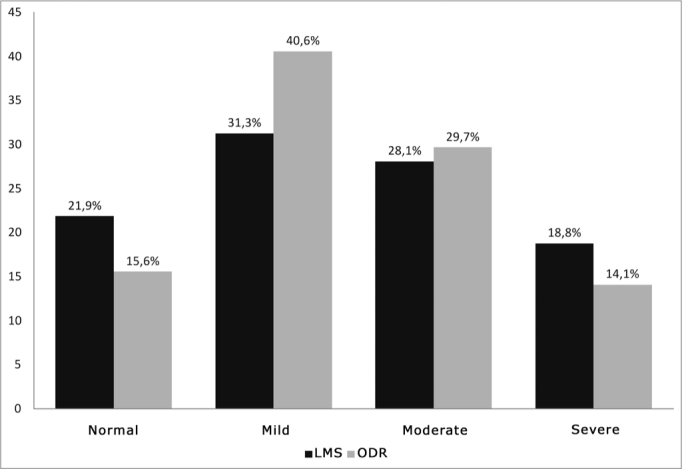
Distribution of the sample per category for each score (%). - LMS: Lund-Mackay system; ODR: opacification/development ratio

The prevalence of exams with abnormalities was 78.1% (LMS) and 74.4 (ODR). Scoring of four sets of readings ranged from 0 to 88, mean 21.3 (SD = 23.1), for the ODR. The variation was 0 to 20, mean 5.2 (SD = 5.3), for the LMS. Chart 1 shows the sample distribution by category, according to each score.

Table 1 shows the intra and interobserver agreement indices based on the ODR and LMS Kappa (k) coefficients, based on a classification by category. The ODR intraobserver was calculated using a single value per exam, which was extracted from the arithmetic mean of two readings for each examiner. The same was done for the LMS. Table 2, Table 3 show the variability calculations of score measurements based on the intra and interclass coefficients (inter-CC and intra-CC),^27^ which correspond respectively to the intraobserver and interobserver variability. In this test, the reproducibility coefficient (r) is an estimate of the maximum difference that could be obtained between two random measures of the same subjects; it is the limit within which are 95% of the differences. The intra-CC r was not more than 4.2 (0 to 24 scale) for the LMS; it was not more than 15.4 (0 to 100 scale) for the ODR. The inter-CC r was 4.0 (LMS) and 15.6 (ODR).

**Table 1:** Intraobserver and interobserver agreement of paranasal sinus opacification categories according to the Lund-Mackay system (LMS) and the opacification/development ratio (ODR) (Kappa values), separated independently for each category and for the general classification (p < 0.001).

Agreements							
		Intraobserver				Interobserver	
		ODR^a^		LMS^a^			
		LFF^b^	SAN^b^	LFF^b^	SAN^b^	ODR^a^	LMS^a^
	Normal	0,81	0,84	0,62	0,91	0,60	0,78
Category	Mild	0,68	0,74	0,58	0,76	0,60	0,63
	Moderate	0,78	0,77	0,75	0,77	0,79	0,57
	Severe	0,94	0,93	0,79	0,94	0,93	0,80
General		0,78	0,80	0,68	0,83	0,71	0,68

a LMS: Lund-Mackay system; ODR: opacification/development ratio

b LFF and SAN: examiners

**Table 2:** Intraobserver measurement variability (1^st^ versus 2^nd^ reading)* for the Lund-Mackay system (LMS) and the opacification/development ratio (ODR).

Examiner	Score	Patients	Measures	Intra-CC (I.C. 95%)	r#
LFF^a^	LMS	64	128	0,93 (0,90 – 0,96)	4,2
	ODR	64	128	0,95 (0,92 – 0,97)	15,4
SAAN^b^	LMS-	64	128	0,97 (0,95 – 0,98)	2,3
	ODR-	64	128	0,98 (0,96 – 0,99)	9,4

* one-way random model ANOVA

# Reproducibility coefficient

a,b Examiners

**Table 3:** Interobserver measurement variability according to the Lund-Mackay system (LMS) and the opacification/development ratio (ODR). The arithmetic mean of two readings by each examiner were calculated for each method to reach a final classification value.

Score	Patients	Measures	Inter CC (C.I.a 95%)	r#
LMS	64	128	0,93 (0,83 – 0,96)	4,0
ODR	64	128	0,95 (0,90 – 0,97)	14,6

*two-way mixed model ANOVA

# Reproducibility coefficient a: Confidence interval

**Chart 2:**
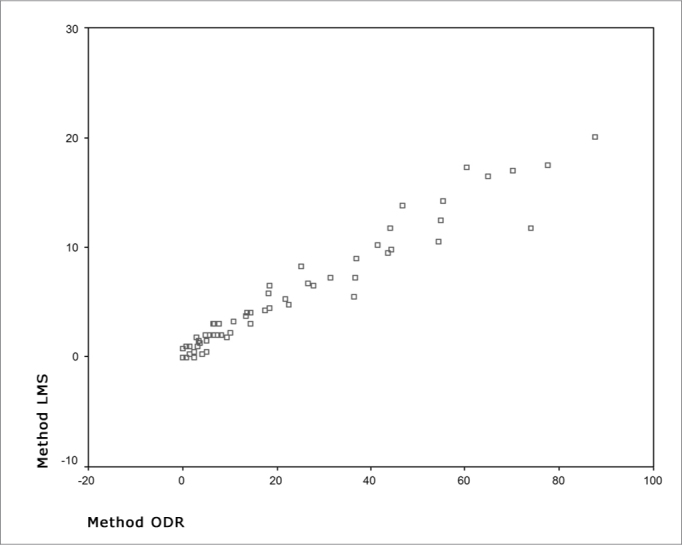
Linear regression between scales of two scores

LMS: Lund-Mackay system; ODR: opacification/development ratio. Pearson's coefficient (r = 0.97). LMS = 0.22 × ODR + 0.43 (r2 = 0.95).

Each exam was represented by a single ODR value and a single LMS value extracted from the mean of four readings (two by each examiner) for the correlation between methods. The methods agreed substantially for categorizing the disease (normal, mild, moderate and severe), where k = 0.68 for the examiner L.F.F., and k = 0.76 for the examiner S.A.A.N (p < 0.001). Chart 2 shows the linear correlation between ODR (0 to 100) and LMS (0 to 24) quantitative scales. Pearson's coefficient revealed a strong linear correlation (r = 0.97) between methods. The ODR score may be converted to its LMS equivalent with the formula: LMS = 0.22 × ODR + 0.43 (r2 = 0.95).

**Chart 3:**
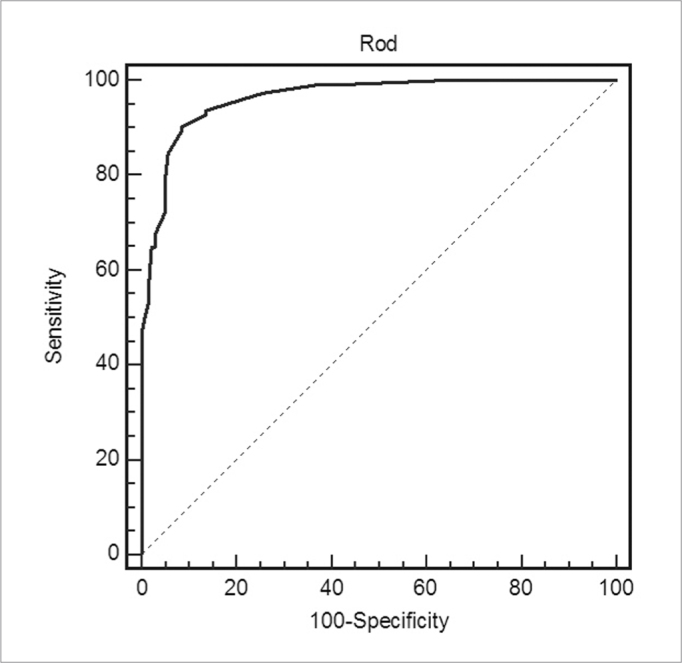
Receiver Operator Characteristic curve for the opacification/ development ratio. Lund-Mackay system (LMS) as the reference test (positive = 4). - ODR: opacification/development ratio

ODR accuracy was calculated taking the LMS as a reference test (positive when the LMS ≥ 4). Inclination of the ROC curve (Chart 3) indicates good accuracy. Table 4 shows the numbers extracted from the curve, where the best ODR sensitivity and specificity values were between 13 and 16.7.

**Table 4:** Receiver operator characteristic curve (ROC) data for the opacification/development ratio (ODR), with the Lund-Mackay system (LMS) as the reference test.

Scale	Sensitivity (%)	C.I.^a^ 95%	Specificity (%)	CI 95%
>10	93,7	87,4 – 97,4	86,5	79,8 – 91,7
>12	92,8	86,3 – 96,8	86,5	79,8 – 91,7
>13	90,1	83,0 – 94,9	91,5	85,6 – 95,5
>16,7	89,2	81,9 – 94,3	91,5	85,6 – 95,5
>17	84,7	76,6 – 90,8	94,3	89,1 – 97,5
>20	78,4	69,6 – 85,6	95,0	90,0 – 98,0

a: Confidence interval

## DISCUSSION

In the present study, the prevalence of exams with any opacification (74% to 78%) was on average similar to the values reported by other authors in disease populations.^11^^,^^16^^,^^25^^,^^28^^,^^29^

The mean LMS score was 5. Reported numbers in the literature vary depending on the sample population. Studies of patients undergoing surgery for the treatment of CRS - patients for which medical treatment was insufficient - resulted in higher means (9 to 13)^16^^,^^22^ and were more intensely altered30 compared to those shown in Chart 1. On the other hand, results are closer in studies of subjects undergoing primary evaluation of RS. A study with this type of population gathered data from several North-American centers^23^ and found that the LMS mean ranged from 1 (one) to 5 (five). This population was probably more similar to our series, as our inclusion criteria for exams were intentionally deliberately ample, regardless of the intensity or duration of the clinical picture. Thus, many patients were not necessarily FES candidates, and even subjects with mild or self-limited symptoms were likely to be included.

The ODR mean was 21. There are no parameters from diseased populations in the literature for comparison purposes. A single previous study of ODR applied this method to evaluated asymptomatic children undergoing studies of the cranium unrelated to RS to seek incidental sinusal findings.^26^ In this study, the ODR mean was lower (15), as expected for individuals aged 3 years and above, which was similar to our age range.

General ODR inter and intraobserver agreement indices for classifying patients into categories were comparable, and at times superior, to the LMS; k generally remained between 0.6 and 0.9, which is considered as substantial agreement.^31^ Oluwole et al.^20^ found a similar LMS performance (interobserver – 0.72; intraobserver −0.73), which are on average superior to those found using other methods such as in Jorgensen,10 May^13^ and Newman;^14^ their intra and interobserver k variables ranged from 0.34 to 0.66. Analysis of quantitative score scales showed that ODR intra and interclass coefficients had significant intraobserver and interobserver reproducibility values (> 0.90), which were invariably higher than LMS values.

There was a strong linear correlation between methods, suggesting that the ODR responds to sinus opacification intensity similarly to the LMS. There was also substantial agreement in sample categorization when both methods were compared, meaning that the cut-of points classified similarly the intensity of opacification.

Nearly all LMS and ODR correlation showed a trend towards higher agreement coefficients in the “normal” and “severe” categories compared to the mild and moderate categories, suggesting that the limit between intermediate categories may be inherently difficult to establish using CT when writing its report, or that the cut-off points for these categories require fine tuning.

The ODR accuracy was calculated indirectly, taking the LMS as the gold standard (positive ≥ 4). The ROC curve was accurate, with best results in the 13 to 17 ODR range. It should be noted that lower values do not necessarily discard inflammation. Likewise, a positive test should not be used alone to establish a diagnosis of RS. Due correlation with clinical findings should be the most important guiding factor.^4^^,^^32^

The LMS was used here for comparison because it is currently the most widely accepted score in the academic community. Some of its deficiencies, however, have been discussed openly in its recommending text.^18^ It has been suggested that partial opacification could be partitioned into more points, rather than just one, to better differentiate intermediate cases. This same article comments that hypoplastic frontal sinuses in adults should score zero in the LMS. There are not references by the LMS authors about what to do in cases of undeveloped sinuses in children. Scoring zero for undeveloped sinuses, was done in a study of children in Oceania,^33^ certainly does not solve underestimation of disease.

Although FES has fewer indication in children and adolescents compared to adults, and is used as the last measure in chronic refractory cases, this procedure has yielded satisfactory results in this age group,^34^ which underlines the importance of an appropriate tomographic score.

None of the currently used scoring systems provide a final result that conveys an idea of opacified area proportion, as the ODR. A few generate categorical variables

(groups),^9^^,^^11^^,^^13^^,^^15^ while others - Jorgensen,^10^ Newman et al.,^14^ and the Miami University method^30^ - are similar to the LMS; they apply points according to the degree of opacification in each sinus. Because of their features, incomplete development may affect all of these systems.

Some authors have studied incidental tomographic findings in the PNS of children without sinus disease and written their own quantification criteria of sinus opacification; these contain the idea of proportion for opacification. Manning et al.^35^ attributed opacification intensity categories in degrees, but the final result was given as classes, which did not translate the total area of disease involvement. Lesserson et al.^36^ and Diament et al.^37^ applied similar criteria, but made a similar decision when presenting the final score (classes).

Because it is a representation of opacification percentages, we expect that ODR results are less affected by the number or size of developed cavities, compared to the LMS. Subsequent studies should include a sufficiently large sample aged below 12 years to confirm this hypothesis. Our sample of subjects with incompletely developed sinuses was small and did not allow consistent statistics.

Our objectives did not require prospective control of diagnostic criteria for RS, as it did not involve correlating tomographic and clinical findings. The exam sample served only for repeated measures of tomographic abnormalities to provide data so that we could assess inter and intraobserver variations of the ODR. In this context, it is desirable to include a wide range of clinical states, from oligosymptomatic to rich clinical pictures, so that the full amplitude of scores is represented, from zero (normal exam) to maximum degrees of opacification. ODR accuracy (sensitivity and specificity) was measured based on LMS as a reference for the same sample. Thus, it is unlikely that lack of data would have affected our results.

Nevertheless, signs and symptoms are currently used as the best parameter for diagnosing RS and measuring the response to therapy. Thus, it is essential for subsequent studies to directly evaluate the accuracy of ODR relative to clinical findings; this will require a diseased population and controls (possibly with CT of the orbit, which is technically similar to examination of the PNS). In this study design, signs and symptoms should be controlled prospectively and with rigor, preferably using a clinical scoring systems. Other parameters may be used as references, such as endoscopic findings or analysis of sinus puncture material.

Patients operated previously were not excluded, as anatomical peculiarities were not expected to affect ODR inter or intraobserver variability; it would be desirable to separate these patients in a sample of prevalence studies or when clinical or surgical data are used as parameters.

## CONCLUSION

The opacification/development ratio (ODR) is an accurate method for evaluating rhinosinusitis in children and adolescents; it is precise and correlates strongly with the Lund-Mackay system (LMS).

The ODR may predict the LMS value with the formula LMS = 0.22 × ODR + 0.43.

The sensitivity and specificity of an ODR value of 15 as a cut-of point for a positive test was close to 90%, based on the LMS as the gold standard.
